# Annotations

**Published:** 1907-01-26

**Authors:** 


### ANNOTATIONS.

### Tropical Diseases Research Fund.

THE Advisory Committee of the Tropical Diseases Research Fund was constituted, in July 1904, by the Secretary of State for the Colonies, and the report of the committee for 1906 has just been issued. The revenue of the Fund for the year amounted to £3,000, and consisted of contributions from the Imperial Government, the Government of India, the Rhodes trustees, and a number of the Colonial Governments. From this sum grants have been made to various organisations concerned in the study and investigation of tropical diseases. Thus a grant of £1,000 has been given to the London School of Tropical Medicine towards the remuneration of teachers and investigators of protozoology and helminthology; and the Liverpool School has received £500 for the payment of a lecturer on economic entomology and parasitology and for the establishment of a lecturer on tropical medicine. Again, a contribution of £750 to the University of London has helped in the foundation of a professorship of protozoology, to which Mr. E. A. Minchin, who was recently engaged in Uganda in the investigation of sleeping sickness, has been appointed; and a grant of £500 has been made to the Royal Society for the purpose of special research work. By all these means the committee has endeavoured to secure the provision of efficient instruction for medical officers, to be employed in the colonies and protectorates, and the promotion of investigation into the nature and causes of tropical diseases. When the history of modern medicine comes to be written there will be few chapters more impressive than those concerned with the extraordinary developments of our knowledge of tropical maladies. One of the most striking features of this movement has been the combination of scientific enthusiasm and ingenuity with practical organising ability. Investigations have been conducted under the most difficult conditions, but have ever been disciplined by the demands of informed criticism. And the whole work has been dominated by practical instincts of the highest order.

### Food and Science.

THE general public has during recent years been treated to many announcements and discussions on the subject of foods and their values. The chemist, the physiologist, the physician, the faddist, have, each alike and in turn, descended from their respective eminences to teach through popular columns and magazine articles the cultivation of the art of feeding. Each doubtless has gained his reward in the shape of a consciousness of duty performed and truth proclaimed. Each, too, it may be expected, has received tributes of admiration and gratitude from a more or less numerous body of disciples, and it may be that in some instances fees or other substantial tokens have not been wholly wanting. Certainly a number of special and individual doctrines bearing on foods in relation to health on the one hand and disease on the other, have attracted a share of public sympathy, as, indeed, may be discovered in the ordinary intercourse of social life. It is not an uncommon experience to meet persons who talk with great confidence and with much pseudo-scientific display of the virtues of some particular form of diet, and of the pernicious consequences which will inevitably attend any departure from the rules within which they enclose themselves. And it cannot be denied that some of these eccentricities owe their origin to medical patronage or even initiation. Yet, after all, it may be questioned whether there is a sufficient scientific basis on which to rest any very definite and exact doctrine in relation to foods, and more particularly as these concern the maintenance of health. Physiological observations and laboratory experiments are of great interest and of some value, but they do not, and cannot, include all the conditions of healthy and vigorous life. Man has learned, by experience extending through long ages, food habits which have had their share in placing him in his present position, and he will hardly be wise suddenly to abandon the results of this experience on the suggestion of calculations which necessarily omit some of the most important activities of human existence.

### Dearth of Young Practitioners.

IT will be seen from a statement in another column, that, hospital managers have found themselves in a difficulty of late, when they have had to fill the resident medical officers' posts. There is a dearth of candidates for these offices, and some of the county hospitals are finding that the qualifications of those who apply are not up to the standard. An attempt is being made to improve the quality of the candidates in the provinces by offering larger salaries. For two generations, at least, the house surgeon, at the majority of such hospitals, has received £100 a year, in addition to maintenance and residential quarters. This salary is being raised twenty-five per cent., and we shall be glad to have information as to whether the increased emolument has attracted more and better candidates for these posts. One reason given for the increasing difficulty in obtaining suitable candidates, for residential posts in hospitals, is the decrease in the number of students. This decrease is believed to be due to the action taken by general practitioners, all over the country, to make it known that, the excessive free hospital relief has so reduced their incomes as to make general practice anything but a profitable occupation for a gentleman of education. It costs the parents of students, or the candidates themselves to obtain a complete medical education and the highest qualifications, about £1,000 in money. When qualified, the men who desire to practise as consultants, and to hold hospital appointments, have to provide for an expenditure of probably more than £2,000 to provide for years of unremunerative labour. No more eloquent testimony than this shortage could be borne to the urgent necessity for the managers of every voluntary hospital altering the present system and restricting the number of those who at present receive free treatment in all departments of these charities.

